# Smoking and Risk of All-cause Mortality: The Jichi Medical School (JMS) Cohort Study

**DOI:** 10.2188/jea.15.173

**Published:** 2005-09-27

**Authors:** Fumihiro Uno, Shizukiyo Ishikawa, Yosikazu Nakamura, Tadao Gotoh, Naoki Nago, Kazunori Kayaba, Eiji Kajii

**Affiliations:** 1 Tako Central Hospital.; 2 Division of Community and Family Medicine, Division of General Practice, Center for Community and Family Medicine, Jichi Medical School.; 3 Department of Public Health, Jichi Medical School.; 4 Wara National Health Insurance Hospital.; 5 Yokosuka General Hospital Uwamachi.; 6 School of Health and Social Services, Saitama Prefectural University.

**Keywords:** Smoking, Mortality, Japan, Cohort Studies

## Abstract

BACKGROUND: There have been comparatively few large-scale cohort studies analyzing all-cause mortality due to cigarette smoking. The goal of this analysis is to investigate the relationship between smoking status and all-cause mortality, and to evaluate the effect of smoking in the Japanese.

METHODS: The baseline data were collected between 1992 and 1995. Ultimately, 10,873 Japanese (4,280 males and 6,593 females) aged 19 years or older from 12 rural communities located across Japan participated in the study. This analysis is based on the results, including the information on those who died and moved out of the communities, obtained by December 31, 2001. The Cox’s proportional hazards model was used to calculate the hazard ratio (HR) of mortality for smoking with adjustment for age, systolic blood pressure, total cholesterol, body mass index, alcohol drinking habit and education.

RESULTS: The mean follow-up period was 8.2 years, during which time, 284 males and 192 females died. The multivariate-adjusted HRs for total mortality among former and current smokers compared with never smokers were 1.09 (95% confidence interval [CI]: 0.73-1.61) and 1.65 (95% CI: 1.16-2.35) in males, and 0.98 (95% CI: 0.40-2.42) and 0.91 (95% CI: 0.42-1.95) in females, respectively. Those for the consumption of 1-14, 15-24, and 25+ cigarettes per day among male smokers were 1.62, 1.57, and 1.89, respectively. In females, there was no great difference in all-cause mortality between smokers and never smokers.

CONCLUSIONS: The results of our study confirm an increased risk in males of premature death from all causes among Japanese with a smoking habit.

The influence on health of smoking is mainly established in Western countries and many studies have pointed out that smoking is a major contributor to mortality from a variety of conditions, such as coronary heart disease, malignant neoplasms, and chronic obstructive lung diseases.^1^^-^^9^ Some papers^10^^-^^19^ evaluated the cause-specific mortality of smoking. On the other hand, there have been comparatively few reports on an increase in all-cause mortality due to cigarette smoking.^20^^-^^28^ Only the limited number of cohort studies in which smoking-attributable mortality rates were calculated have been conducted in Japan.^29^^-^^31^ In particular, many researches are carried out for people who reside in the limited areas recently. Even though the proportion of smokers decreased gradually for a few decades,^32^ the harmful effect of smoking has actually shown a reversal in recent years. We found it difficult to generalize the results reported in the past to the present situation. In order to promote the health of the general population, the effect on total mortality is more important than that on cause-specific mortality. We investigated the mortality due to smoking and evaluated the effect of smoking in the large number of population-based cohort subjects. It would be worth verifying whether or not there is any discrepancy with the results of previous studies.

## METHODS

The aim of the Jichi Medical School (JMS) Cohort Study was to investigate the risk factors of cardiovascular disease in Japan. The study design and some descriptive data have been presented previously.^33^ This research was conducted on residents who underwent a basic medical checkup. Residents aged 40-69 years were the subjects of the mass screening examination program in 8 of the 12 communities and those aged 30 years and older in one community. Subjects from other age groups were included in the remaining communities.

The baseline data were obtained from 1992 through 1995. To obtain information using a uniform method, we established a central committee, which was composed of the chief medical officers of all the participating districts. The committee developed a detailed manual for data collection. Body mass index (BMI) was calculated as weight (kg) divided by the square of height (m). The systolic and diastolic blood pressures were measured with a fully automated sphygmomanometer, BP203RV-II (Nippon Colin, Komaki, Japan). Total cholesterol levels were measured by an enzymatic method (Wako, Osaka, Japan; inter assay coefficient of variation [CV]: 1.5 %).

Information about the medical history and life style was obtained using a questionnaire to confirm whether or not the subject was taking medication and determine the existence of past or present illnesses. When the questionnaire was incomplete, the blank items were confirmed directly by trained interviewers. The questionnaire items relating to smoking determined whether or not the subject was a current or former smoker, the number of cigarettes smoked per day by current or former smokers, and the year when smoking was started or stopped.

**Figure 1.**
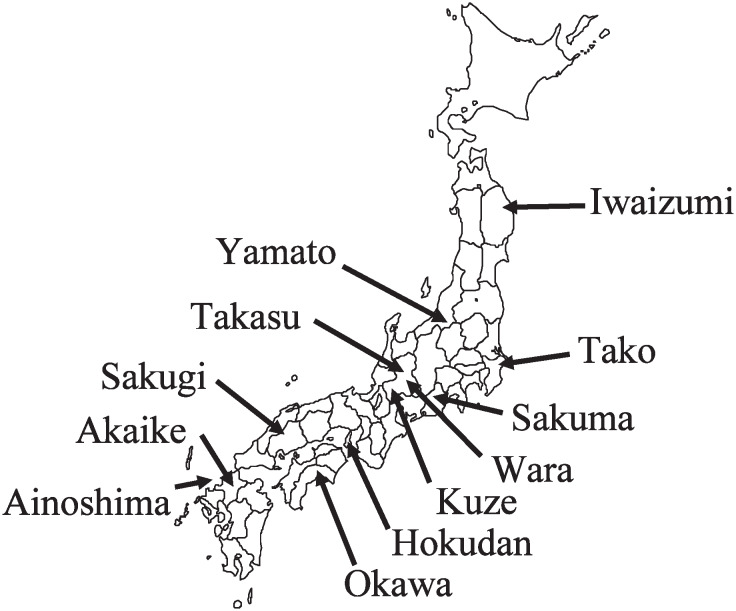
The twelve districts participating in the JMS Cohort Study.

A total of 12,490 persons (4,911 males and 7,579 females) from 12 rural communities (see Figure 1) located across Japan participated in the study and the response rate for the residents who underwent a basic medical checkup was about 99%. The rate of people who actually underwent a medical checkup for the total population was 65.4%.^33^ In this analysis, we excluded 664 persons who had a history of stroke, myocardial infarction, or malignant neoplasms as determined by a questionnaire, and 953 persons from whom the information on smoking habits was incomplete. As a result, the final number of study subjects was 4,280 males aged between 18 and 90 years and 6,593 females aged between 19 and 89 years in the 12 communities. In each community, the local government office sent letters to all potential participants inviting them to take part in the program. Death certificates were collected at public health centers with the permission of the Ministry of Public Management, Home Affairs, Posts and Telecommunications and the Ministry of Health, Labour and Welfare. Data on emigrants among the study subjects were obtained by each municipal government annually. There were 369 emigrants at the time of analysis. The emigrants were treated as candidates for analysis until the time of transfer and these data were regarded as censored. We started to follow up the subjects from the final year when the base line data were acquired in each district. This analysis is based on the results obtained by December 31, 2001 when the information on those who died, had events and moved out of the communities was confirmed. As a result, the mean follow-up period was 8.2 years for the cohort subjects, and 284 male and 192 female deaths from all causes were confirmed from the death certificates.

Statistical analysis was performed using the SAS^®^ 8.2 edition (SAS institute, Inc., Cary, North Carolina, USA). Descriptive parameters are shown as mean, standard deviation or percentile. The Cox’s proportional hazards model^34^ was used to calculate the hazard ratios (HRs) of mortality for smoking after adjusting for age, systolic blood pressure, total cholesterol level, body mass index, educational background and habit of drinking alcohol at baseline.

Smoking status was judged as current smoking, former smoking or never, and if currently smoking, the number of cigarettes consumed per day was recorded. Current smokers were further graded into 1-14, 15-24, and 25+ cigarettes per day. A former smoker was defined as a person who had given up smoking at the time of baseline data acquisition, but who had smoked previously. Alcohol drinking status was classified as current drinking, former drinking or never drinking. Educational level was defined by the age at final education. Age at final education was categorized as age<15, 15-17, and 18+ years.

The crude mortality ratios were calculated per 1,000 person-years. Population attributable fraction (PAF) was calculated as Pd×(RR-1)/RR where Pd = proportion of cases exposed to the risk factor, i.e., the combined proportion of current and past smokers.^35^ This formula is known to be more valid than the popular form (RR-1)×Pe/{1+(RR-1)×Pe}, where Pe = proportion of source population exposed to the risk factor, when confounding variables exist.^35^

## RESULTS

Table 1 shows the characteristics of the subjects at baseline. The tendency for the age to be young was observed among current smokers of both sexes. Age, blood pressure, and body mass index of both sexes were similar. Total cholesterol levels in females were higher than those in males. In relation to the alcohol drinking status, the proportion of current drinkers among males was higher than that among females.

**Table 1.** General characteristics of the JMS Cohort Study subjects at baseline.

	Males						Females		
	
	Never smoker	Former smoker	Current smoker (number of cigarettes consumed per day)				Never smoker	Former smoker	Current smoker

			All	1-14	15-24	25+			
Pearson-years	7,462	9,684	17,950	3,108	9,701	5,018	49,495	1,509	3,059
No. of subjects	910	1,181	2,189	379	1,183	612	6,036	184	373
No. of death	43	76	165	39	91	33	178	5	9
Mean age (year)	55.3 ± 11.7	58.2 ± 10.7	52.9 ± 12.4	57.9 ± 10.8	54.0 ± 12.2	47.8 ± 11.0	55.6 ± 10.8	48.0 ± 15.7	49.0 ± 13.4

Age (year, %)									
<19	4 ( 0.4)	0 ( 0.0)	5 ( 0.2)	2 ( 0.5)	2 ( 0.2)	1 ( 0.1)	1 ( 0.0)	0 ( 0.0)	2 ( 0.6)
20-29	19 ( 2.1)	0 ( 0.0)	150 ( 6.7)	33 ( 8.1)	29 ( 2.5)	88 ( 13.1)	89 ( 1.5)	24 ( 13.0)	28 ( 7.8)
30-39	49 ( 5.4)	68 ( 5.8)	239 ( 10.6)	27 ( 6.7)	112 ( 9.5)	100 ( 14.9)	388 ( 6.4)	40 ( 21.7)	48 ( 13.4)
40-49	189 ( 20.8)	198 ( 16.8)	557 ( 24.7)	50 ( 12.3)	277 ( 23.5)	230 ( 34.3)	1,211 ( 20.1)	40 ( 21.7)	115 ( 32.0)
50-59	283 ( 31.1)	267 ( 22.6)	455 ( 20.2)	65 ( 16.0)	256 ( 21.7)	134 ( 20.0)	1,767 ( 29.3)	27 ( 14.7)	69 ( 19.2)
60-69	313 ( 34.4)	546 ( 46.2)	759 ( 33.7)	196 ( 48.4)	448 ( 38.0)	115 ( 17.1)	2,309 ( 38.3)	37 ( 20.1)	84 ( 23.4)
70-79	37 ( 4.1)	89 ( 7.5)	78 ( 3.5)	25 ( 6.2)	50 ( 4.2)	3 ( 0.4)	234 ( 3.9)	15 ( 8.2)	13 ( 3.6)
80+	16 ( 1.8)	13 ( 1.1)	12 ( 0.5)	7 ( 1.7)	5 ( 0.4)	0 ( 0.0)	37 ( 0.6)	1 ( 0.5)	0 ( 0.0)

Systolic blood pressure (mmHg)	132.5 ± 20.7	133.5 ± 19.7	129.2 ± 20.5	129.2 ± 20.7	129.7 ± 20.9	128.3 ± 19.6	128.4 ± 20.8	121.3 ± 19.9	120.5 ± 22.1
Diastolic blood pressure (mmHg)	80.0 ± 12.1	80.6 ± 11.9	77.8 ± 12.4	77.6 ± 11.9	78.1 ± 12.5	77.5 ± 12.5	76.4 ± 12.0	73.3 ± 12.0	72.9 ± 12.8
Total cholesterol (mg/dL)	186.4 ± 33.4	189.5 ± 32.3	182.1 ± 35.6	175.9 ± 33.9	182.2 ± 35.3	185.6 ± 36.8	197.4 ± 34.6	188.2 ± 36.1	186.7 ± 34.9
Body mass index (kg/m^2^)	23.4 ± 3.0	23.1 ± 2.8	22.7 ± 2.9	22.0 ± 2.7	22.6 ± 2.8	23.1 ± 3.1	23.2 ± 3.2	22.5 ± 3.0	22.4 ± 3.5

Alcohol drinking status (%)									
Never drinker	235 ( 27.2)	230 ( 19.9)	402 ( 19.0)	77 ( 20.8)	224 ( 19.6)	101 ( 16.7)	4,387 ( 75.5)	85 ( 46.4)	178 ( 50.4)
Former drinker	20 ( 2.3)	71 ( 6.1)	47 ( 2.2)	7 ( 1.9)	30 ( 2.6)	10 ( 1.7)	63 ( 1.1)	15 ( 8.2)	11 ( 3.1)
Current drinker	608 ( 70.5)	855 ( 74.0)	1,671 ( 78.8)	286 ( 77.3)	890 ( 77.8)	495 ( 81.7)	1,362 ( 23.4)	83 ( 45.4)	164 ( 46.5)

Age (year) at finished education (%)									
<15	123 ( 13.6)	174 ( 14.8)	228 ( 10.6)	63 ( 16.8)	136 ( 11.6)	29 ( 4.8)	1,236 ( 20.6)	36 ( 19.7)	62 ( 17.7)
15-17	479 ( 53.1)	609 ( 51.9)	1,093 ( 50.8)	198 ( 52.7)	602 ( 51.5)	293 ( 48.3)	2,898 ( 48.4)	55 ( 30.1)	155 ( 44.2)
18+	300 ( 33.3)	390 ( 33.2)	831 ( 38.6)	115 ( 30.6)	431 ( 36.9)	285 ( 47.0)	1,856 ( 31.0)	92 ( 50.3)	134 ( 38.2)

Mean values±standard deviations for Mean age, Systolic blood pressure, Diastolic blood pressure, Total cholesterol, Body mass index.

The crude mortality rates according to the smoking status are given in Table 2. Observation on the daily number of cigarettes was not conducted for females because the number of deaths was few among former and current female smokers. The crude mortality rates among males were 5.8 per 1,000 person-years for never smokers, 7.8 for former smokers, and 9.2 for current smokers. In females, the corresponding rates were 3.6, 3.3 and 2.9, respectively.

**Table 2.** Adjusted hazard ratios by smoking habits.

Sex		No. of subjects	No. of death	Crude mortality rate (/1,000 person-years)	HR-age* (95% confidence interval)	HR-all^†^ (95% confidence interval)
Males	Never smoker	910	43	5.8	1.00 (reference)	1.00 (reference)
	Former smoker	1,181	76	7.8	1.09 (0.75-1.59)	1.09 (0.73-1.61)
	Current smoker	2,189	165	9.2	1.76 (1.26-2.45)	1.65 (1.16-2.35)
	No. of cigarettes consumed per day among current smokers					
	1-14	379	39	12.5	1.76 (1.14-2.71)	1.62 (1.03-2.56)
	15-24	1,183	91	9.4	1.71 (1.19-2.45)	1.57 (1.07-2.30)
	25+	612	33	6.6	2.05 (1.29-3.24)	1.89 (1.17-3.07)
					P for trend < 0.001	P for trend < 0.001

Females	Never smoker	6,036	178	3.6	1.00 (reference)	1.00 (reference)
	Former smoker	184	5	3.3	1.10 (0.41-2.47)	0.98 (0.40-2.42)
	Current smoker	373	9	2.9	1.16 (0.59-2.26)	0.91 (0.42-1.95)
					P for trend = 0.84	P for trend = 0.80

* : Age-adjusted hazard ratio.

† : Adjusted hazard ratio for age in years; systolic blood pressure in mmHg (less than 140, 140-159, or 160 or higher); total cholesterol in mg/dL (less than 160, 160-199, 200-239, 240-279, or 280 or higher); body mass index in kg/m2 (less than 20.0, 20.0-22.9, 23.0-25.9, or 25 or higher); habit of drinking alcohol (never drinkers, former drinkers, current drinkers); educational background in years of age (up to 16, 16-18, or 19 or older).

In Table 2, age and multivariate-adjusted HRs of all-cause mortality in males and females by smoking categories are shown. Using never smokers as the reference, the age-adjusted HR of male former smokers was 1.09 (95% confidence interval [CI]: 0.75-1.59), and that of current smokers was 1.76 (95% CI: 1.26-2.45). In females, the age-adjusted HR of former smokers was 1.10 (95% CI: 0.41-2.47), and that of current smokers was 1.16 (95% CI: 0.59-2.26). After adjusting for age, systolic blood pressure, total cholesterol level, body mass index, educational background and habit of drinking alcohol at baseline, the corresponding multivariate-adjusted HRs were 1.09 (95% CI: 0.73-1.61), and 1.65 (95% CI: 1.16-2.35) in males, and 0.98 (95% CI: 0.40-2.42), and 0.91 (95% CI: 0.42-1.95) in females, respectively. The HRs also were estimated according to the number of cigarettes consumed per day at baseline, and the results are presented. The dose-response relationship was observed between the number of the cigarettes and HR. The PAFs of smoking for males were 24.9%.

## DISCUSSION

In this study, we have shown that smoking elevates the mortality among male smokers from all causes of death, even after adjustment for some potentially confounding factors. The age-adjusted HRs for male former smokers and current smokers were higher relative to never smokers. Furthermore, a dose-response relationship was observed among male smokers. We also calculated the HRs of mortality for smoking after adjusting for age, systolic blood pressure, total cholesterol level, body mass index, educational background and habit of drinking alcohol at baseline. It was thought that these participated in death directly as confounding factors. In these multivariate-adjusted analyses, the same relationship was also observed among these factors. On the contrary, the crude mortality rates decreased as the number of cigarettes smoked increased. This finding might be attributable to the fall in the average age in proportion to the number of cigarettes smoked.

Therefore, it was considered that age was the most important confounding factor. On the other hand, the difference in all-cause mortality between female smokers and never smokers was not great. This might be attributable to the small total number of female deaths. Although not described in Table 1, there were only 14 persons who smoked 25+ cigarettes per day. This also might have influenced the female results. Depending on the area, some subjects were less than 40 years of age, which might have influenced the results and weakened the HRs of mortality for smoking. However, analysis of the age group limited to 40+ years did not yield a significantly different result, hence the findings of analysis of the whole subject population were used.

In Japan, Hirayama first reported the relative risks (RRs) of mortality due to smoking.^29^ This six prefecture cohort study (SPCS) followed up 122,261 males and 142,857 females aged 40 years and over during the period between 1966 and 1981. The SPCS reported the age-adjusted RRs of the total mortality ranging from 1.29 (90% CI: 1.26-1.32) in male smokers and 1.31 (90% CI: 1.27-1.36) in female smokers. As Lee et al. have pointed out, there were some problems in the cohort study described in their report,^36^ and the time background was different from that of the current study. It also was a problem that subjects with a history of stroke, myocardial infarction, or malignant neoplasms were not excluded from the SPCS analyses. As our recent study had a different background and methodological advantage compared with the six prefecture cohort study, it is not appropriate to compare both studies by numerical means alone.

Hara et al. reported the multivariate-adjusted HR for total mortality of 1.66 (95% CI: 1.40-1.95) in male smokers in their Japan Public Health Center-based Prospective Study (JPHCS) following up 19,950 males and 21,534 females aged 40-59 years during 1990-1999.^30^ The Miyagi Cohort Study (MCS) followed up 36,052 subjects aged 40-64 years during an 11-year period,^31^ and the multivariate-adjusted RRs were 1.71 in males and 1.44 in females. The results of these two studies for males approximated ours and a comparison was reasonable because their research was similar to ours in terms of subjects, observation time, and period of observation.

The PAFs of smoking were 24.9% for males in this study. Our study yielded a low value for females as well as the HRs of mortality, so the calculation of PAF was omitted. The corresponding results were 22.2% in JPHCS and 34% in MCS, respectively. In males, a major difference in results was not found among our and these two studies.

In Western and Asian countries, some reports have estimated the RRs of smoking-attributable mortalities. In the Cancer Preventive Study II in the United States,^5^ the RR due to smoking for all-cause mortality was reported to be 2.3 in males and 1.9 in females. Tang et al. reported in their cohorts in the United Kingdom,^20^ that the multivariate-adjusted RRs of male current smokers were 1.68 (plain-cigarettes), 2.23 (filter-cigarettes), and 2.34 (other). In China, Lam et al. reported that the RRs of current smokers were 2.42 for males and 2.32 for females.^21^ These results were higher than those of the present study. Other reports showed that the RRs of current smokers were higher than our results.^22^^-^^26^ On the other hand, some reports showed almost equal or lower values relative to our results.^27^^,^^28^ The male RRs of these reports are summarized in Table 3.

**Table 3.** Adjusted relative risks (RRs) in other studies (males).

Studies	Adjustment^†^	Person-years	RR(Former smoker)	RR(Current smoker)
Foreign countries				
Cancer Preventive Study II^5^	age	2,981,526	not available	2.30*
The Established Populations for Epidemiologic Studies of the Elderly^22^	age, community	11,892	1.50*	2.10*
California Retirement Community^23^	age	35,640	1.24*	1.95*
The prospective study of four British cohorts^20^	age	731,315	1.23*	1.68*-2.34*
The Prospective Male Cohort Study in Shanghai, China^27^	age, alcohol habit	98,517	1.40*	1.20*-1.60*
The Copenhagen Center for Prospective Population Studies^26^	age	255,000	1.30***	1.90*-2.60*
The cohort analytic study in a machinery factory in Xi'an, China^21^	age, DBP,TG, TC, marital status, occupation, education	21,283	not available	2.42*
The Seven Countries Study^28^	age, TC, SBP, BMI	319,000	1.10	1.30*-1.80*

Japan				
The six-prefecture cohort study^29^	age	1,709,273	not available	1.29*
The Miyagi Cohort Study^31^	age, BMI, education, marital states, walking time, dietary habit, alcohol habit	194,720	1.38*	1.71*
The Japan Public Health Center-based Prospective Study^30^	age, BMI, HT history, area, education, medication, dietary habit, alcohol habit	193,321	1.02	1.66*
The Jichi Medical School Cohort Study^33^	age, SBP, TC, BMI, education, alcohol habit	35,096	1.09	1.65*

† : SBP: systric blood pressure, DBP: diastric blood pressure, TG: triglyceride, TC: total cholesterol, BMI: body mass index, HT: hypertention

* : P<0.05, ***: P<0.001

The kind of cigarettes, the rate of smoking, and the age at which smoking was started varied among these reports. It is considered that the conduct and method of smoking also differed. Although it is difficult to compare simply, it seems that there are many reports with higher RRs of mortality due to smoking in Western countries than in Japan. It was suggested that the study subjects in Japan might have been exposed to a lower dose of smoking because of the low supply of cigarettes after World War II.^30^^,^^36^ Though it has a point in this hypothesis, this view is not supported by the fact that no great difference exists in the mortality between China,^21^ which also experienced cigarette shortages, and Western countries.^20^^,^^22^^-^^26^ It appears difficult to explain the difference in the results of these researches consistently. If there is variation among these results, it might derive from ethnic differences. Further comparison by accumulating future report results is recommended.

The strong points of our research are as follows. First, the study population was defined as all inhabitants covering the whole country from the north to the south of Japan. There are few cohort studies in which smoking-attributable mortality rates have been calculated in Japan since the 1990s. It is thought that accumulation of data on smoking and death is useful for future epidemiological studies. It would be worth verifying whether there is any adjustment with the results of past research. Second, we adjusted for possible confounders such as age, systolic blood pressure, total cholesterol levels, body mass index, habit of drinking alcohol and educational background, and conducted this research with an adequately accurate method of measurement. Accordingly, we aimed at thorough unification of the methodology, such as blood pressure measurement and blood testing. In order to eliminate variation arising from individual techniques, the same automatic sphygmomanometer was used in all areas. Furthermore, the blood samples were analyzed at a single trusted laboratory using the same method of measurement. Accordingly, we believe that the reliability of the data will be high.

The limitation of our research is that the smoking status was determined only at baseline. It is possible that the influence of smoking is undervalued by this problem because the proportion of smokers decreased gradually for a few decades.^32^ However, this problem is common to other large cohort studies, and a study in which the smoking situation is pursued is exceptional. And, in the present investigation, it was difficult to analyze female mortality because there were very few deaths among female smokers.

In conclusion, the results of our study confirm an increased risk of premature death from all causes among Japanese with a smoking habit. Vigorous efforts to assist individuals to abstain from smoking should be stressed as an integral part of primary care.
